# Cottonseed-derived gossypol and ethanol extracts differentially regulate cell viability and VEGF gene expression in mouse macrophages

**DOI:** 10.1038/s41598-021-95248-4

**Published:** 2021-08-03

**Authors:** Heping Cao, Kandan Sethumadhavan, Xiaoyu Wu, Xiaochun Zeng

**Affiliations:** 1 U.S. Department of Agriculture, Agricultural Research Service, Southern Regional Research Center, New Orleans, LA 70124 USA; 2 School of Bioscience and Bioengineering, Jiangxi Agricultural University, Nanchang, 330045 Jiangxi Province China; 3 Department of Life Science and Environmental Resources, Yichun University, Yichun, 336000 Jiangxi Province China

**Keywords:** Biochemistry, Plant sciences, Biomarkers

## Abstract

Vascular endothelial growth factor (VEGF) plays an important role in chronic inflammation associated with several diseases. Many plant extracts have nutritional and healthy benefits by down-regulating VEGF expression, but there was no report on VEGF regulation by cottonseed extracts in any biological system. The objective was to investigate cell viability and VEGF expression regulated by gossypol and ethanol extracts using lipopolysaccharides (LPS) as a control. MTT, qPCR and immunoblotting techniques were used to monitor cell viability, VEGF mRNA and protein levels in mouse RAW264.7 macrophages. Gossypol dramatically reduced macrophage viability but cottonseed extracts and LPS exhibited minor effect on cell viability. VEGFb mRNA levels were approximately 40 fold of VEGFa in the macrophages. Gossypol increased VEGFa and VEGFb mRNA levels up to 27 and 4 fold, respectively, and increased VEGF protein. LPS increased VEGFa mRNA by sixfold but decreased VEGFb mRNA. LPS increased VEGF protein in 2–4 h but decreased in 8–24 h. Glanded seed extracts showed some stimulating effects on VEGF mRNA levels. Glandless seed coat extract showed increased VEGFb mRNA levels but its kernel extract reduced VEGF mRNA levels. This study demonstrated that gossypol and ethanol extracts differentially regulated cell viability and VEGF expression in mouse macrophages.

## Introduction

Vascular endothelial growth factor (VEGF) is a major adipogenesis mediator^1^ and a mitogenic and angiogenic factor critical for inflammation, tumor progression, collateral vessel formation, and diabetic retinopathy^2^. VEGFa and VEGFb isoforms play a balance role in adipose differentiation and gene expression. VEGF expression is regulated by a number of agents including bacteria-derived endotoxin lipopolysaccharides (LPS) and insulin. It was reported that LPS increases VEGF expression in mouse RAW264.7 macrophages^3^. Pulmonary expression of the VEGF family and their receptors is down-regulated in LPS-induced lung injury^4^. Insulin increases anti-inflammatory tristetraptolin (TTP) and decreases proinflammatory VEGF gene expression in mouse adipocytes^5^.

Plant extracts and compounds with lowering VEGF expression activities could have a positive effect on nutrition and health. Green tea-derived polyphenolic epigallocatechin gallate (EGCG) pre-treatment suppresses LPS-induced inflammatory response and oxidant stress and exerts its hepatocyte-protective activity by reducing the production of VEGF and other cytokines in LPS-stimulated hepatocytes^6^. Cinnamon extract reduces VEGF mRNA in the cultured adipocytes^7^ and mice^8^, and inhibits angiogenesis in zebrafish and human endothelial cells^9^. Triphala herbal extract suppresses inflammatory responses by decreasing the production of inflammatory mediators including VEGF in LPS-stimulated RAW264.7 macrophages and adjuvant-induced arthritic rats^10^. In contrast, Camellia oil (*Camellia oleifera* Abel.) increases VEGF gene expression in ketoprofen-damaged gastrointestinal mucosal tissue^11^. These studies indicate that VEGF expression is up- or down-regulated by plant extracts depending on the source of extracts. Therefore, this area of research is deserved for more studies.

**Figure 1:**
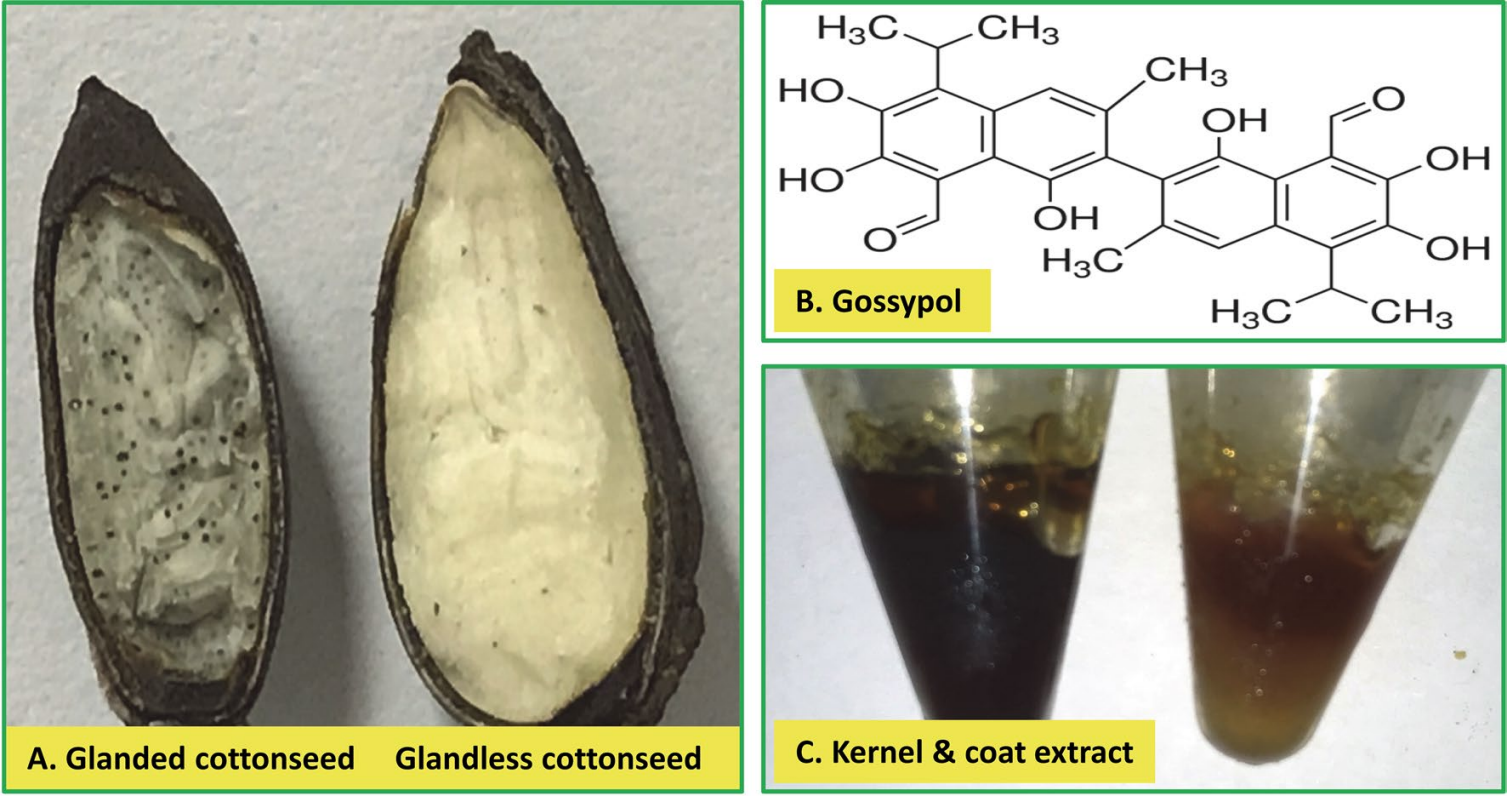
Cottonseed, gossypol, and ethanol extracts. (**A**) Cottonseed. Glanded and glandless cottonseeds are indistinguishable outside but glanded cottonseed is smaller than glandless cottonseed and contains numerous dark green-colored glands. (**B**) Structure of gossypol isolated from cottonseed (Image was from Sigma product sheet). (**C**) Cottonseed ethanol extracts.

Cottonseed is classified as glanded or glandless seed depending on the presence of the gossypol glands (Fig. 1A)^12–15^. Cottonseed contributes to approximately 20% of the cotton plant (*Gossypium hirsutum* L.) value^16^. Commercial cottonseed meal contains approximately 1% of gossypol^17^, which limits its use primarily to feed ruminants^18–22^. Glandless cottonseed lacks pigment glands and has trace levels of gossypol which may be useful for potential utilization of the protein as a food ingredient or as a feed for non-ruminant animals^23–26^. It is possible to increase cottonseed value by extracting bioactive extracts and compounds for health promotion and disease prevention^27–30^. Cottonseed contains many bioactive components including gossypol^31^, gallic acid and 3,4-dihydroxybenzoic acid^32^, bioactive peptides^33–35^, flavonol glycosides^32^, and quercetin^36^.

Gossypol is a plant polyphenol in the pigment glands of cotton (Fig. 1B)^31^. Gossypol and similar compounds have anti-cancer activities associated with breast cancer^37^, colon cancer^38^, pancreatic cancer^39^ and prostate cancer^40^. They have anti-obesity^37^, anti-inflammatory^41^, and anti-fungal activities^42^. It was reported that gossypol inhibited VEGF expression in human breast cancer cells, resulted in both apoptosis and anti-angiogenesis effects^43^. Gossypol treatment causes different responses in cytotoxicity and angiogenic cytokine secretion of the two hormone- and drug-resistant prostate cancer cell lines^44^. These findings have generated much interest in biomedical research for potential medical utilization of gossypol and related compounds.

We recently isolated bioactive ethanol extracts essentially free of gossypol from cottonseed (Fig. 1C)^28^. These cottonseed extracts affect human cancer cell growth^28^ and regulate diacylglycerol acyltransferase (DGAT) and anti-inflammatory tristetraprolin (TTP/ZFP36) gene expression in mouse macrophages^27,30^, but has minimal effect on the expression of human antigen R (HuR), a protein stabilizing some cytokine mRNAs involved in tumorigenesis and inflammation^45^. There was no prior research if cottonseed extracts can regulate VEGF gene expression in any biological system.

We hypothesized that cottonseed-derived gossypol and ethanol extracts, like green tea EGCG and cinnamon extract, might down-regulate VEGF gene expression in mouse macrophages since they are plant polyphenols and polyphenolic extracts isolated from cottonseed and cinnamon bark by similar methods^28,46,47^. Therefore, the objective of this study was to assess the effect of gossypol and ethanol extracts from cottonseed on cell viability and regulation of VEGF gene expression using endotoxin LPS as a control since LPS is a strong inflammation stimulator^48,49^ in mouse RAW264.7 macrophages, a preferred cell model for inflammation research^50^, in which VEGF was shown modestly regulated by plant extracts like cinnamon polyphenolic extract^51^. However, contrary to our hypothesis, our results showed that gossypol strongly decreased cell viability, but cottonseed extracts had only minor effects on its viability. qPCR and immunoblotting assays showed that gossypol increased VEGF mRNA and protein in macrophages and that glanded seed extracts modestly increased VEGF mRNA levels, but that only glandless cottonseed kernel extract down-regulated VEGF mRNA levels. This study demonstrated that cottonseed-derived gossypol and ethanol extracts differentially regulated cell viability and VEGF expression in mouse macrophages.

## Results

### Effect of gossypol, LPS and cottonseed extracts on macrophage viability

**Figure 2:**
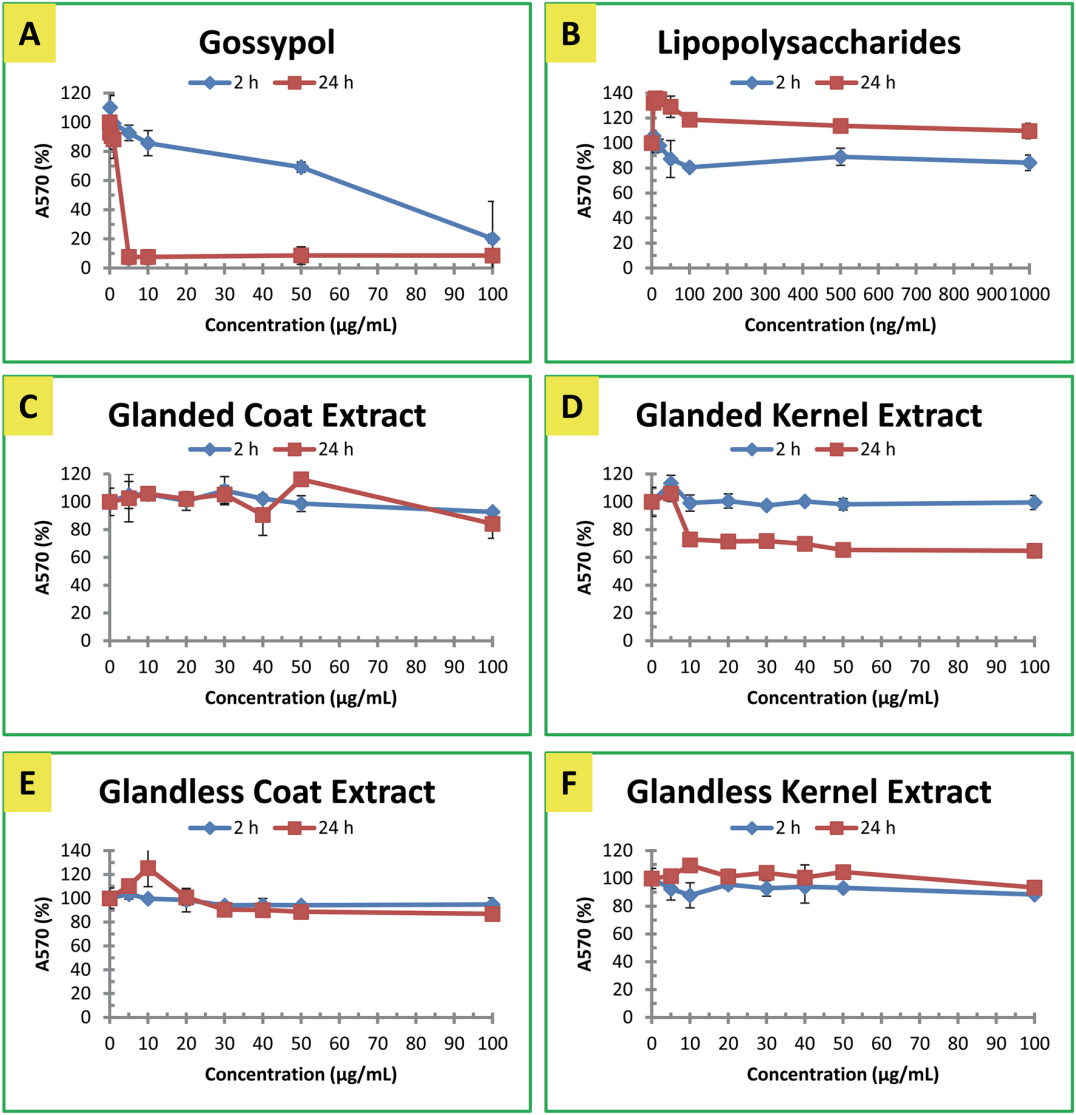
Effect of Gossypol, LPS and cottonseed extracts on mouse macrophage viability. (**A**) Gossypol, (**B**) LPS. (**C**) Glanded cottonseed coat extract, (**D**) Glanded cottonseed kernel extract, (**E**) Glandless cottonseed coat extract, (**F**) Glandless cottonseed kernel extract. RAW264.7 macrophages were treated with cottonseed extracts for 2 and 24 h. Macrophage viability was determined by MTT assay. The data represent the mean and standard deviation of three independent samples.

Macrophage viability was determined by MTT method after cells were treated with various concentrations of the agents for 2 and 24 h (Fig. 2). Gossypol exhibited significant inhibitory effect over the DMSO control on macrophage viability under high concentration or long treatment time (Fig. 2A). LPS exhibited positive effect on macrophage viability under low concentration treatment for 24 h (Fig. 2B). Glanded cottonseed coat extract did not affect the viability of macrophages (Fig. 2C). Glanded cottonseed kernel extract decreased macrophage viability by about 30% after 24 h treatment (Fig. 2D). Extracts from coat (Fig. 2E) and kernel (Fig. 2F**)** of glandless cottonseed did not have significant effect on cell viability after 2–24 h treatment.

### Relative abundance of VEGF mRNAs in mouse macrophages

To provide a basis for the comparison of VEGF gene expression, the relative mRNA levels of two VEGF genes were measured in the mouse RAW264.7 macrophages treated with 1% DMSO control by qPCR using the specific primers (Table 1). The qPCR assay showed that VEGFb mRNA levels were much more abundant than VEGFa in macrophages (Table 2). SYBR Green qPCR showed that VEGFb mRNA levels were 33, 36 and 52 fold of VEGFa in the cells collected at 2, 8 and 24 h, respectively (Table 2). To confirm SYBR Green qPCR results, TaqMan qPCR was also performed and the results showed that VEGFb mRNA levels were approximately 20–40 fold higher than VEGFa in the macrophages (Table 2). These results confirmed that both SYBR Green and TaqMan qPCR assays are reliable for determining gene expression^52,53^. SYBR Green qPCR was chosen to conduct further gene expression analysis for cost saving and convenience.

**Table 1:** Nucleotide sequences information of qPCR primers.

mRNA	Accession no	Amplicon (bp)	Forward primer (5′–3′)	TaqMan probe (5′–3′)	Reverse primer (5′–3′)
Vegfa	NM_001025250	68	CAAAAACGAAAGCGCAAGAAA	CCCGGTTTAAATCCTGGAGCG	CGCTCTGAACAAGGCTCACA
Vegfb	NM_011697	83	GATCCAGTACCCGAGCAGTCA	TGTCCCTGGAAGAACACAGCCAATGTG	TCTCCTTTTTTTTTGGTCTGCAT
Rpl32	NM_172086	66	AACCGAAAAGCCATTGTAGAAA	AGCAGCACAGCTGGCCATCAGAGTC	CCTGGCGTTGGGATTGG

**Table 2:** Relative expression of VEGFa and VEGFb mRNAs in mouse macrophages.

qPCR method	Time	mRNA	C_T_ ± SD (*n*)	Fold (2^−ΔΔCT^)*
SYBR	2 h	Rpl32	18.16 ± 0.71 (6)	
		Vegfa	30.75 ± 1.45 (7)	1.00
		Vegfb	25.43 ± 0.57 (6)	32.72
	8 h	Rpl32	19.12 ± 0.94 (5)	
		Vegfa	33.92 ± 2.38 (7)	1.00
		Vegfb	28.45 ± 1.71 (4)	36.30
	24 h	Rpl32	17.82 ± 0.81 (5)	
		Vegfa	32.07 ± 0.95 (5)	1.00
		Vegfb	26.07 ± 1.05 (5)	52.34
TaqMan	0 h	Rpl32	18.19 ± 0.04 (4)	
		Vegfa	29.02 ± 0.34 (4)	1.00
		Vegfb	24.00 ± 0.17 (4)	32.39
	0.5 h	Rpl32	18.17 ± 0.10 (4)	
		Vegfa	28.71 ± 0.44 (4)	1.00
		Vegfb	23.96 ± 0.15 (4)	26.77
	1 h	Rpl32	18.10 ± 0.03 (4)	
		Vegfa	29.20 ± 0.10 (4)	1.00
		Vegfb	23.92 ± 0.30 (4)	38.85
	2 h	Rpl32	18.10 ± 0.06 (4)	
		Vegfa	29.05 ± 0.22 (4)	1.00
		Vegfb	23.90 ± 0.07 (4)	35.51
	4 h	Rpl32	18.10 ± 0.10 (4)	
		Vegfa	28.55 ± 0.39 (4)	1.00
		Vegfb	24.26 ± 0.14 (4)	19.56

*Relative fold calculation for SYBR green qPCR: VEGFb/VEGFa mRNA levels = 2^−ΔΔCT^*68 bp (length of VEGFa amplicon)/83 bp (length of VEGFb amplicon).

### Effect of gossypol on VEGF mRNA levels

**Figure 3:**
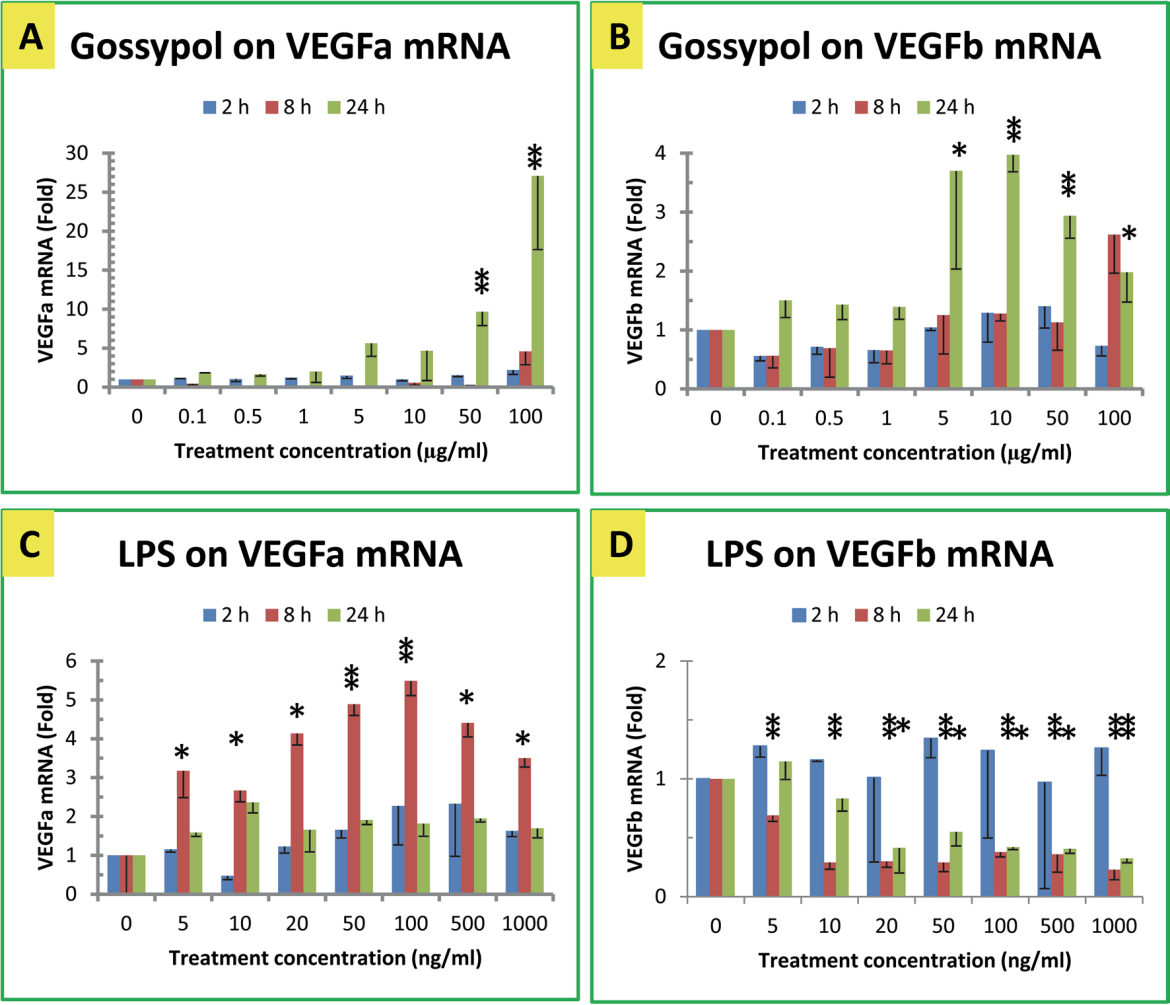
Effect of gossypol and LPS on VEGF gene expression. (**A**) Effect of gossypol on VEGFa mRNA. (**B**) Effect of gossypol on VEGFb mRNA. (**C**) Effect of LPS on VEGFa mRNA. (**D**) Effect of LPS on VEGFb mRNA. RAW264.7 macrophages were treated with gossypol (0–100 µg/mL) or LPS (0–1000 ng/mL) for 2, 8 and 24 h**.** The data represent the mean and standard error of three independent samples. “*” and “**” displayed above each of the treatment time are significantly different between the treatment and the control at *p* < 0.05 and *p* < 0.01, respectively.

Mouse macrophages were treated with different concentrations of gossypol. qPCR assay showed that gossypol gradually increased VEGFa mRNA levels in mouse macrophages as its concentrations increased (Fig. 3A). VEGFa mRNA levels were increased to 5–10 fold of the control with 5–50 µg/mL gossypol and more than 27 fold with 100 µg/mL gossypol after 24 h treatment (Fig. 3A). Gossypol effect on the expression fold of VEGFb mRNA was less than that of its effect on VEGFa mRNA in mouse macrophages (Fig. 3B). VEGFb mRNA levels were increased approximately fourfold with statistical significance by gossypol stimulation at 5–100 µg/mL for 24 h (Fig. 3B). Since basal level of VEGFb mRNA was 30–50 fold of VEGFa mRNA (Table 2), the net increase of VEGFb mRNA was judged to be much more than that of VEGFa mRNA.

### Effect of LPS on VEGF mRNA levels

LPS is an agent that causes inflammation and increases the expression of many genes such as the anti-inflammatory TTP gene^48,51^. The effects of LPS on VEGF gene expression were conducted in RAW264.7 macrophages. LPS significantly induced VEGFa gene expression in mouse macrophages. VEGFa mRNA levels were increased up to fivefold by LPS treatment for 8 h (Fig. 3C). In contrast, VEGFb mRNA levels were significantly decreased in macrophages treated with LPS for 8 h or 24 h (Fig. 3D). Considering the fact that the basal level of VEGFa mRNA was much less than that of VEGFb mRNA (Table 2), it was likely that the net loss of VEGFb mRNA was still larger than the net gain of VEGFa mRNA.

### Effect of glanded cottonseed extracts on VEGF mRNA levels

**Figure 4:**
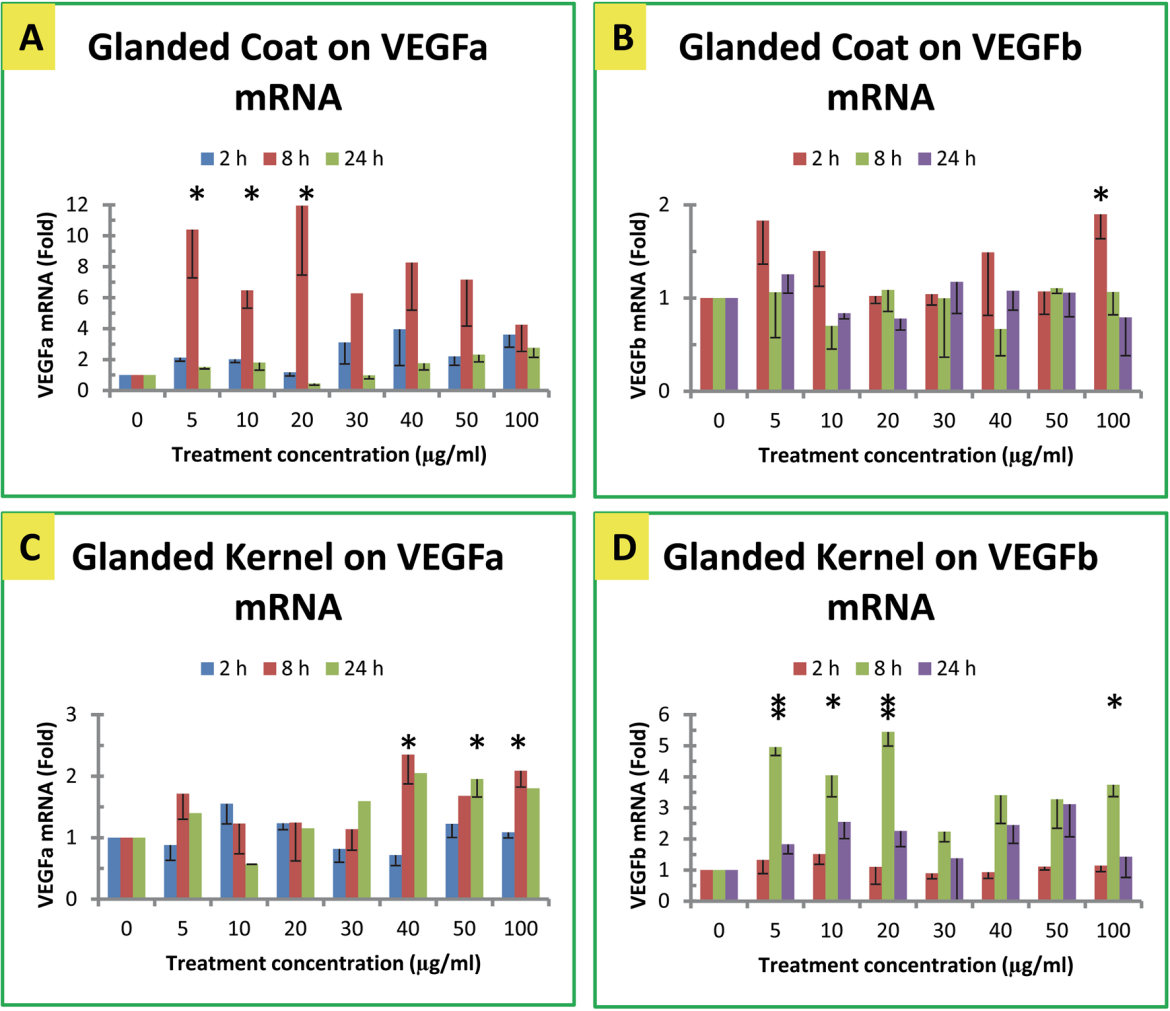
Effect of glanded cottonseed extracts on VEGF gene expression. (**A**) Effect of glanded coat extract on VEGFa mRNA. (**B**) Effect of glanded coat extract on VEGFb mRNA. (**C**) Effect of glanded kernel extract on VEGFa mRNA. (**D**) Effect of glanded kernel exract on VEGFb mRNA. RAW264.7 macrophages were treated with glanded cottonseed coat and kerenl extracts (0–100 µg/mL) for 2, 8 and 24 h. The data represent the mean and standard error of three independent samples.

**Figure 5:**
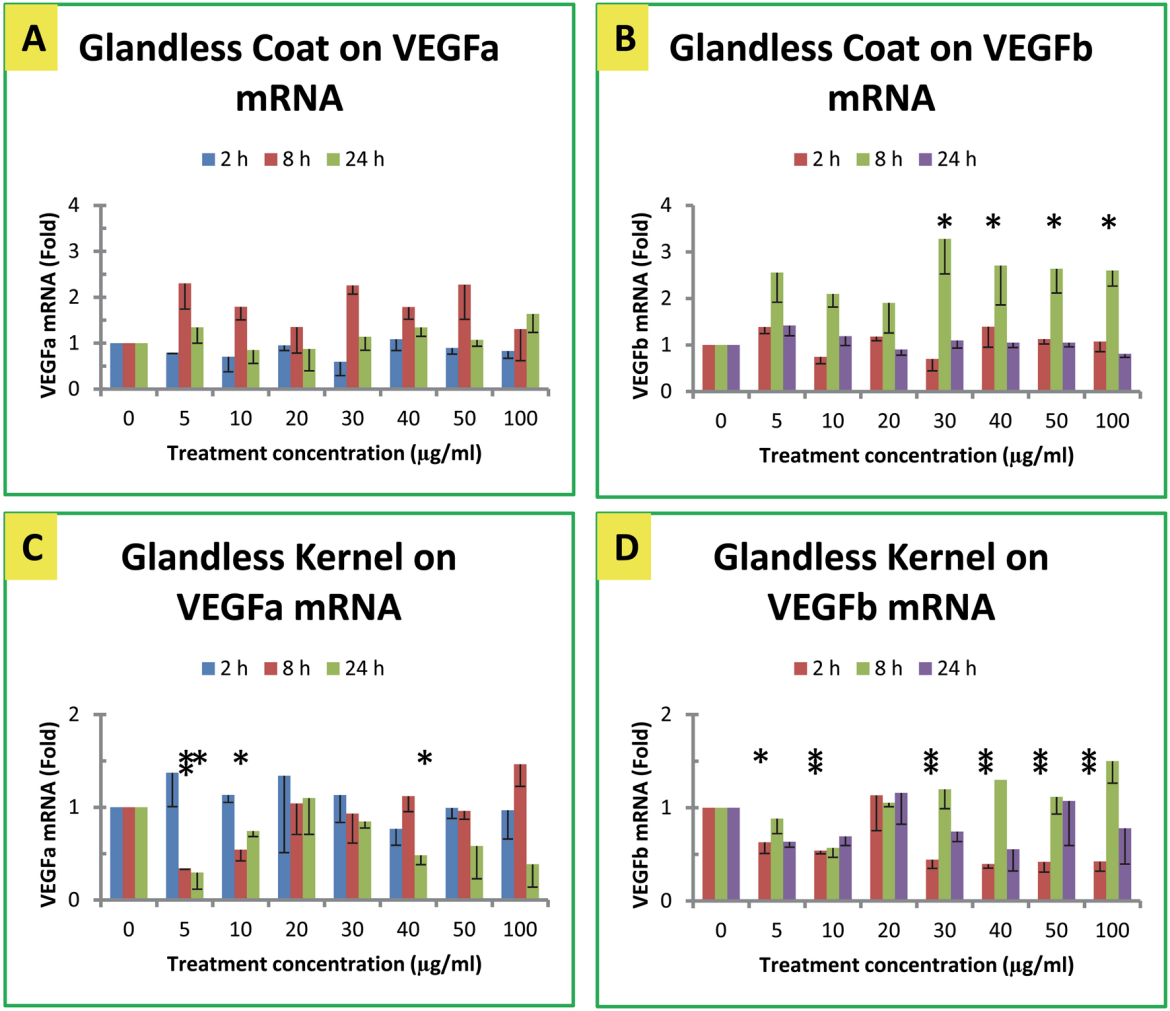
Effect of glandless cottonseed extracts on VEGF gene expression. (**A**) Effect of glandless coat extract on VEGFa mRNA. (**B**) Effect of glandless coat extract on VEGFb mRNA. (**C**) Effect of glandless kernel extract on VEGFa mRNA. (**D**) Effect of glandless kernel extract on VEGFb mRNA. RAW264.7 macrophages were treated with glanded cottonseed coat and kerenl extracts (0–100 µg/mL) for 2, 8 and 24 h. The data represent the mean and standard error of three independent samples.

In general, cottonseed extracts exhibited minor effects on VEGF gene expression in mouse macrophages (Figs. 4, 5). Glanded cottonseed coat extract appeared to increase VEGFa mRNA levels in macrophages after 8 h treatment (Fig. 4A), but exhibited much less effect on VEGFb mRNA levels in macrophages treated for 2, 8 or 24 h under various concentrations (Fig. 4B). Glanded cottonseed kernel extract modestly increased VEGFa mRNA levels (Fig. 4C) and VEGFb mRNA levels in macrophages treated for 8 h (Fig. 4D).

### Effect of glandless cottonseed extracts on VEGF mRNA levels

Glandless cottonseed coat extract modestly increased VEGFa and VEGFb mRNA levels in the macrophages treated for 8 h (Fig. 5A, B). Glandless cottonseed kernel extract significantly decreased VEGFa mRNA levels (Fig. 5C) and VEGFb mRNA levels in the macrophages (Fig. 5D).

### Effect of gossypol, LPS and cottonseed extracts on VEGF protein levels

**Figure 6:**
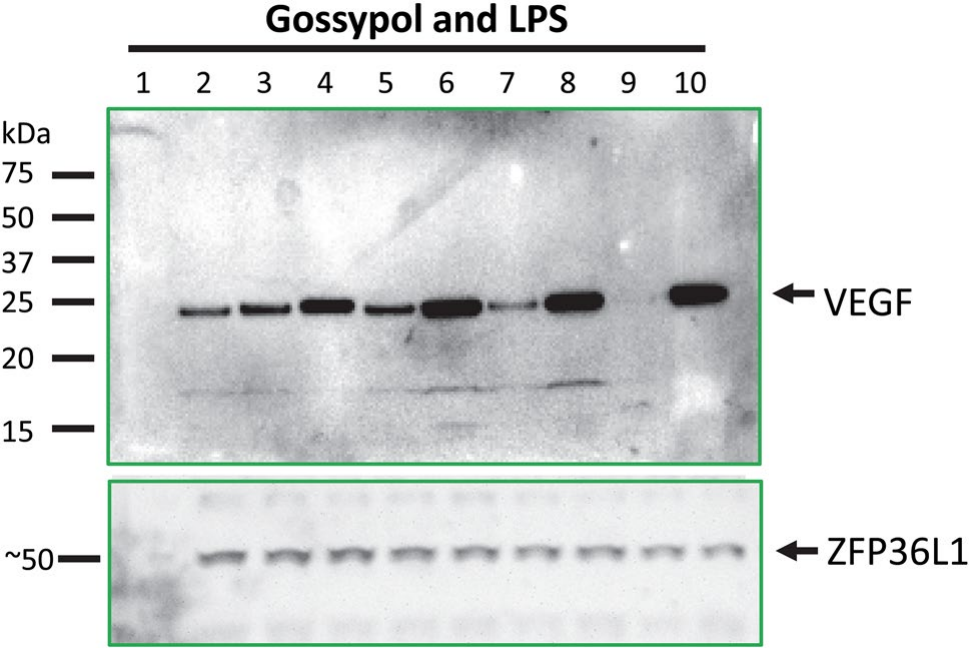
Effect of gossypol and LPS on VEGF protein levels in mouse macrophages. RAW264.7 cells were stimulated with 100 µg/mL gossypol or 100 ng/mL LPS. Cell extract was used for immunoblotting using the anti-VEGF polyclonal antibodies ab46154 and using the ZFP36L1 antibodies for equal loading purpose. Lane 1: protein standards; lane 2: 1% DMSO; lanes 3, 5, 7 and 9: LPS treatment for 2, 4, 8 and 24 h, respectively; lanes 4, 6, 8 and 10: gossypol treatment for 2, 4, 8 and 24 h, respectively.

**Figure 7:**
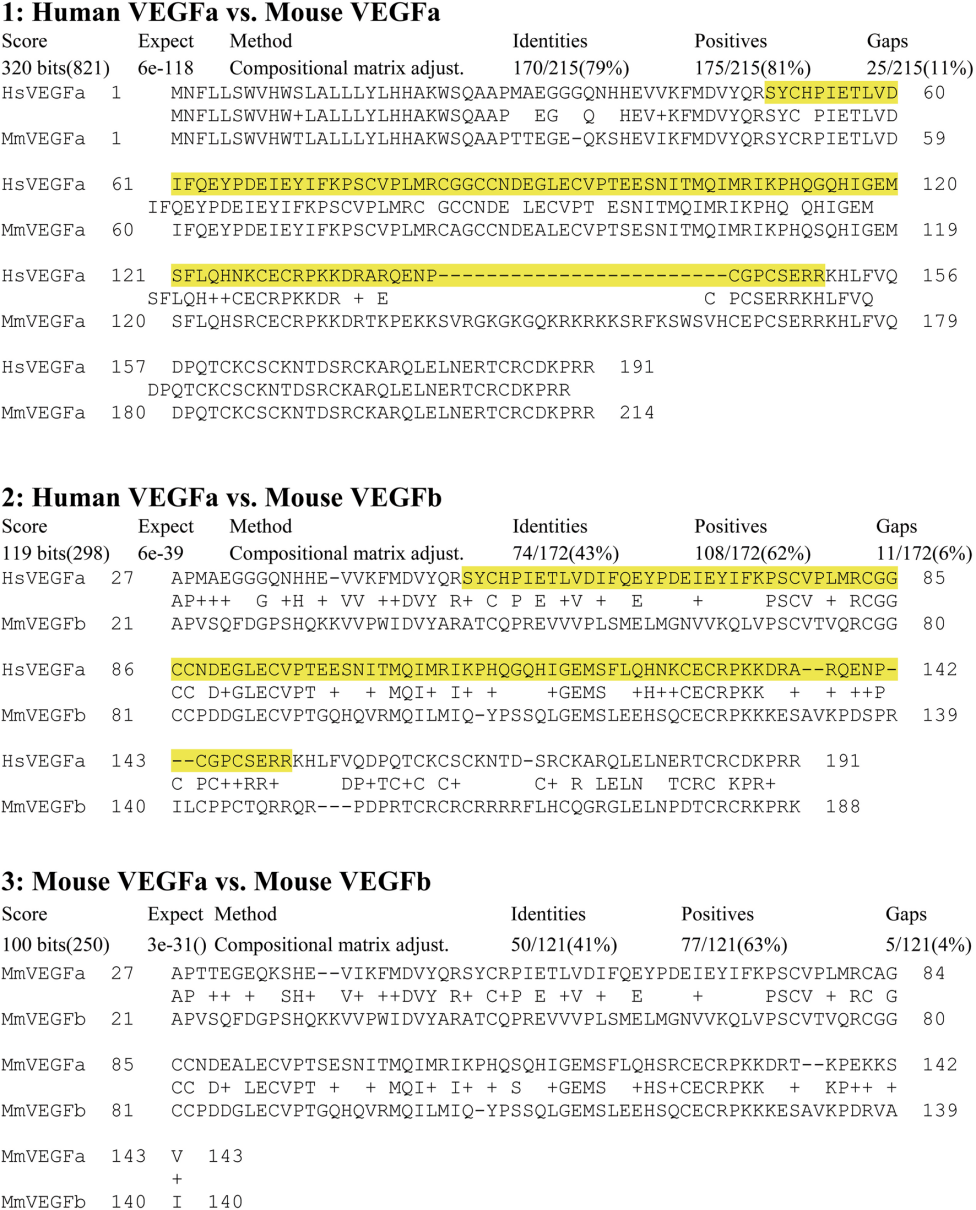
Amino acid sequence alignment between human VEGFa and mouse VEGFa and VEGFb proteins. VEGF polyclonal antibodies (ab46154) used in the study were raised in rabbits against a human VEGFa peptide corresponding to amino acid residues 50–150 as highlighted in yellow color.

Immunoblotting was used to determine if VEGF protein levels were affected by gossypol, LPS and cottonseed extracts in mouse macrophages (Fig. 6 and data not shown). Anti-VEGF antibodies detected a band of approximately 24 kDa, which corresponded to the predicted size of VEGF protein in DMSO-controlled cells (Fig. 6, lane 2). Gossypol strongly increased VEGF protein levels in the cells treated at 100 µg/mL for 2, 4, 8 and 24 h (Fig. 6, lanes 4, 6, 8 and 10 vs. lane 2). It appeared that VEGF protein levels were increased more in cells treated by gossypol for longer time than 2 h (Fig. 6, lanes 6, 8 and 10 vs. lane 4). LPS-treated macrophages at 100 ng/mL showed darker VEGF antibody-reactive bands than the control with similar size in cells treated for 2 and 4 h (Fig. 6, lanes 3 and 5 vs. lane 2), but the intensity was decreased in 8 and 24 h (Fig. 6, lanes 7 and 9 vs. lane 2), probably due to reduction of VEGFb mRNA (Table 2), the major form of VEGF mRNAs, by LPS treatment (Fig. 3D). VEGF polyclonal antibodies (ab46154) used here were raised in rabbits against a human VEGFa peptide corresponding to amino acid residues 50–150. Sequence comparison indicated that human VEGFa is 79% identical to mouse VEGFa and 43% identical to mouse VEGFb (Fig. 7). The intensity of immune-reactive peptides in LPS-treated samples more closely corresponded to VEGFb mRNA levels. It might be due to the fact that VEGFb mRNA levels were 20–40 fold higher than VEGFa mRNA levels in macrophages (Table 2), although it is unknown how the polyclonal antibodies derived from human VEGFa cross-reacted with mouse VEGFa and VEGFb proteins differentially.

Glanded cottonseed coat and kernel extracts did not show apparent effect on VEGF protein levels in macrophages treated at 100 µg/mL for 2, 4, 8 and 24 h (data not shown). VEGF protein levels were not affected neither by glandless cottonseed coat or kernel extracts in macrophages treated at 100 µg/mL for 2, 4, 8 and 24 h (data not shown). The undetectable difference in protein levels compared to qPCR data was probably due to the less sensitivity of immunoblotting than qPCR assay. It is not always easy to correlate mRNA levels and protein levels in the cells. Besides the relative abundance of mRNA molecule, there are many additional variables from mRNA transcription to protein translation such as mRNA stability, mRNA translocation from nucleus to the cytosol, post-transcriptional modification, mRNA degradation, and accumulation of excessive mRNA molecules into stress granules.

## Discussion

VEGFs are growth factors for endothelial cells involved in inflammation, tumor progression, collateral vessel formation, and diabetic retinopathy^2,54–56^. VEGFb is proposed to be a strong anti-oxidant^57^. Both VEGFa and VEGFb proteins shared 41% of amino acid sequence identity (Fig. 7) but little identity at the nucleotide sequence level (data not shown). VEGFa and VEGFb play a balance role in adipose differentiation, gene expression and function in energy metabolism^58^. Plant compounds that can down-regulate VEGF expression may have nutritional and therapeutic value. In this study, we tested the effect of cottonseed-derived gossypol and ethanol extracts on cell viability and regulation of VEGFa and VEGFb gene expression in mouse macrophages using LPS as a control. The results suggest that gossypol and ethanol extracts differentially regulated cell viability and VEGF expression in mouse macrophages.

Our results showed that cottonseed-derived gossypol and ethanol extracts differentially regulated cell viability in mouse macrophages. MTT assays showed that cottonseed-derived gossypol exhibited significant inhibition on macrophage viability; gossypol at higher concentration or longer treatment caused more reduction of mitochondrial activity. In contrast, the extracts from the coat and kernel of glanded and glandless cottonseed did not have significant effect on cell viability after treatment for 2–24 h with up to 100 µg/mL of the extracts in the culture medium. The results presented here that gossypol inhibited cell viability are different from a previous study^41^. The discrepancy between these two studies is unclear. It is possible due to cell density used in the two studies: the previous study used 4 × 10^5^ cells/mL in 96-well plate and ours used ¼ of their cells in 24-well plate. It is also possible due to the final concentrations of gossypol used in both studies^41^. The cytotoxic compound gossypol is known to be accumulated in the glanded cottonseed which causes male infertility^19^ but minimally present in glandless cottonseed^26^. Our results suggest that gossypol may affect immunity when over-consumption and accumulation of this toxic compound in the body. The cottonseed extracts are essentially free of the toxic compound gossypol with only 0.82, 0.03, 0.37 and 0 ng of gossypol per mg of the extracts from glanded coat, glanded kernel, glandless coat and glandless kernel, respectively^28^. These results suggest that cottonseed extracts are probably safe for consumption.

Our results also showed that cottonseed-derived gossypol and ethanol extracts differentially regulated VEGF gene expression in mouse macrophages by both qPCR and immunoblotting assays. Cottonseed-derived gossypol stimulated VEGFa and VEGFb mRNA levels up to 27 and 4 fold, respectively. Immunoblotting confirmed that macrophage VEGF protein was significantly increased by gossypol. In contrast, cottonseed extracts had small effects on VEGF gene expression in mouse macrophages. Cottonseed extracts from glanded seed exhibited modest stimulation effects on VEGFa and VEGFb mRNA levels in mouse macrophages. Importantly, ethanol extracts from glandless cottonseed kernel appeared to lower the VEGF mRNA levels in mouse macrophages. The lack of stimulation effect by cottonseed extracts on VEGFa and VEGFb expression contrasted to that of the gossypol. These results confirmed HPLC–MS results that ethanol extracts from cottonseed are essentially free of gossypol, in agreement with our previous analysis of gossypol being present in the extracts with less than 1 ppm gossypol, which was much less than the US federal government limit of 450 ppm^29^. However, our results are different from a previous publication where gossypol was shown to decrease VEGF expression in human breast cancer cells^43^. The discrepancy between these two studies may be due to the use of different cell types. Our qPCR results showed that gossypol strongly induced both VEGFa and VEGFb mRNA levels in mouse macrophages. However, gossypol decreased VEGF mRNA levels in human breast cancer cells^43^ and colon cancer cells^59^. It was shown in breast cancer cells that gossypol decreased VEGF mRNA levels indirectly by promoting mRNA degradation via MDM2 RING protein binding to VEGF mRNA molecule^43^. VEGF mRNA levels in colon cancer cells were extremely lower than those in macrophages (approximately 10 C_T_ difference)^59^. Therefore, it is difficult to evaluate the significance of gossypol-down regulating VEGF mRNA levels in the colon cancer cells. It could be important to learn how gossypol increased VEGF mRNA levels in macrophages and the significance of the up-regulation since they are relative abundant mRNA molecules in mouse macrophages.

Bacteria-derived proinflammatory LPS was shown to stimulate VEGF gene expression at both mRNA and protein levels^3,4,60^. In our previous paper^51^, we only tested VEGF expression in mouse macrophages with 10 ng/mL LPS within 4 h treatment. LPS at 10 ng/mL did not affect VEGFa gene expression and decreased VEGFb mRNA levels in cells treated for up to 4 h^51^. In this study, we expanded the study by treating the macrophages with much higher concentration (up to 1000 ng/mL) for much longer time (up to 24 h). We confirmed that LPS stimulated VEGFa mRNA levels up to sixfold with 50–500 ng/mL treatment for 8 h. However, LPS significantly decreased VEGFb mRNA levels in macrophages after 8–24 h treatment. Immunoblotting showed that VEGF protein levels were increased significantly in 2–4 h but declined in 8–24 h by LPS, in agreement with qPCR data showing VEGFb mRNA as the major form was decreased by LPS. This up-down trend of LPS-regulation of VEGF expression is similar to that of LPS-regulation of TTP/ZFP36 expression in mouse macrophages^48^.

## Conclusions

Gossypol had stronger inhibition on cell viability and profound stimulation on VEGF gene expression in the macrophages. Gossypol increased more VEGF mRNA and protein than LPS and the effect was sustained. Ethanol extracts from cottonseed exhibited minor effects on cell viability and VEGF gene expression in mouse macrophages. Ethanol extracts from glandless cottonseed kernel decreased VEGF mRNA levels in mouse macrophages This study suggests that cottonseed-derived gossypol and ethanol extracts differentially regulated cell viability and VEGF expression in mouse macrophages.

## Methods

### Cell line

Mouse RAW264.7 macrophages were from American Type Culture Collection and stored in a cryogenic storage vessel under liquid nitrogen vapor.

### Chemicals and reagents

qPCR primers were designed using Primer Express software and synthesized by Biosearch Technologies (Table 1). Bacteria-derived LPS and dimethylsulfoxide (DMSO) were from Sigma. Cell culture reagents were from Gibco BRL. TRIzol was from Thermo Fisher. cDNA synthesizing reagents were from Life Technologies. SYBR Green Supermix was from Bio-Rad.

### Cottonseed-derived gossypol and ethanol extracts

Cottonseed-derived gossypol was purchased from Sigma. Ethanol extracts of cottonseed were isolated from glanded and glandless cottonseed (Fig. 1A)^28^. The cottonseed extracts contain 0.82, 0.03, 0.37 and 0 ng of gossypol per mg of the extracts from glanded cottonseed coat and kernel, and glandless cottonseed coat and kernel, respectively^28^.

### Cell culture and treatment

Mouse macrophages were maintained at 37 °C with 5% CO_2_ in DMEM containing 4.5 mg/mL (25 mM) glucose, 10% (v:v) fetal bovine serum, 100 units/mL penicillin, 100 µg/mL streptomycin, and 2 mM L-glutamine^48^. RAW cells were subcultured in 24-well plates (0.5 mL, 1 × 10^5^ cells/mL). The chemicals and extracts were dissolved in 100% DMSO and diluted with water to the desired concentrations. Raw macrophages were treated with 5–100 µg/mL of cottonseed extracts, 0.1–100 µg/mL of gossypol, or 5–1000 ng/mL of LPS for 2–24 h (“0” treatment as the control corresponded to 1% DMSO in the culture medium). The dosages of chemicals and ethanol extracts were selected based on our previous study on two other important genes^27,45^.

### Cell cytotoxicity assay

MTT method was used to assess cell viability using the In Vitro Toxicology Assay Kit essentially as described previously^28^. Macrophages(0.5 mL) were treated with cottonseed extracts, gossypol and LPS and incubated at 37 °C, 5% CO_2_ for 2 and 24 h. Thiazolyl blue tetrazolium bromide (50 µL) was added to the medium, and incubated at 37 °C, 5% CO_2_ for 2 h before adding 0.5 mL MTT solubilization solution. The color density was measured at A570 nm by microplate spectrophotometer (Epoch) and SmartSpec plus Spectrophotometer (BioRad). DMSO treatment was used as the control for cell viability assay^28^.

### RNA extraction, cDNA synthesis and real-time qPCR analysis

RNAs were isolated from macrophages using TRIzol reagent^51^. The cDNAs were synthesized from total RNAs as described^51^. The cDNAs were diluted to 1 ng/μL before qPCR analyses. SYBR Green qPCR reaction mixtures contained 5 ng of total RNA-derived cDNAs and the thermal cycle conditions were identical to those described (56). RPL32 mRNA was used as the internal control and DMSO treatment was used as the sample control for qPCR assay as described^27,53^. The ΔΔ*C*_*T*_ method of relative quantification was used to determine the fold change in gene expression (Table 2)^61^.

### Cell extraction, protein determination, SDS-PAGE and immunoblotting

Cell extracts were prepared with a described procedure^48^. Bradford method determined protein concentrations in the 10,000 g supernatant using the Bio-Rad reagent. Proteins (100 µg per lane) were separated by 8–12% SDS-PAGE and transferred onto a PVDF membrane with iBlot Gel Transfer System. The membrane was blocked with 5% nonfat dry milk in TTBS buffer and incubated with anti-VEGF antibody (1:500 in blocking buffer) (Abcam cat# ab46154). Antibodies raised against recombinant mouse ZFP36L1 protein were used for immunoblotting control since ZFP36L1 expression was stable in mouse macrophages^51,62^. After washed with TTBS buffer, the membrane was incubated with affinity-purified goat anti-rabbit IgG (H + L) horseradish peroxidase conjugate (1:5,000 in TTBS buffer). After the membrane was finally incubated with ECL Prime Western Blotting Detection Reagent and chemiluminescent intensity was captured by ChemiDoc Touch Imaging System.

### Statistics

The data represent the mean and standard deviation or standard error of 3 independent samples. ANOVA with SigmaStat 3.1 software was used to analyze qPCR data. Student–Newman–Keuls Method was used to make multiple comparisons among the treatments^7^.

## Supplementary Information

Supplementary Information.

## Data Availability

All relevant data are within the paper.
