# Supplementary material for: Cottonseed-derived gossypol and ethanol extracts differentially regulate cell viability and VEGF gene expression in mouse macrophages
Source: Sci Rep. 2021 Aug 3;11:15700. doi: 10.1038/s41598-021-95248-4 (PMC8333419; doi:10.1038/s41598-021-95248-4)
Supplement: Supplementary file 1 — Supplementary Information. [file 41598_2021_95248_MOESM1_ESM.pdf]

Cottonseed-derived Gossypol and Ethanol Extracts Differentially Regulate Cell Viability and VEGF Gene Expression in Mouse Macrophages

Heping Cao [1,*], Kandan Sethumadhavan [2], Xiaoyu Wu [3] and Xiaochun Zeng[4]

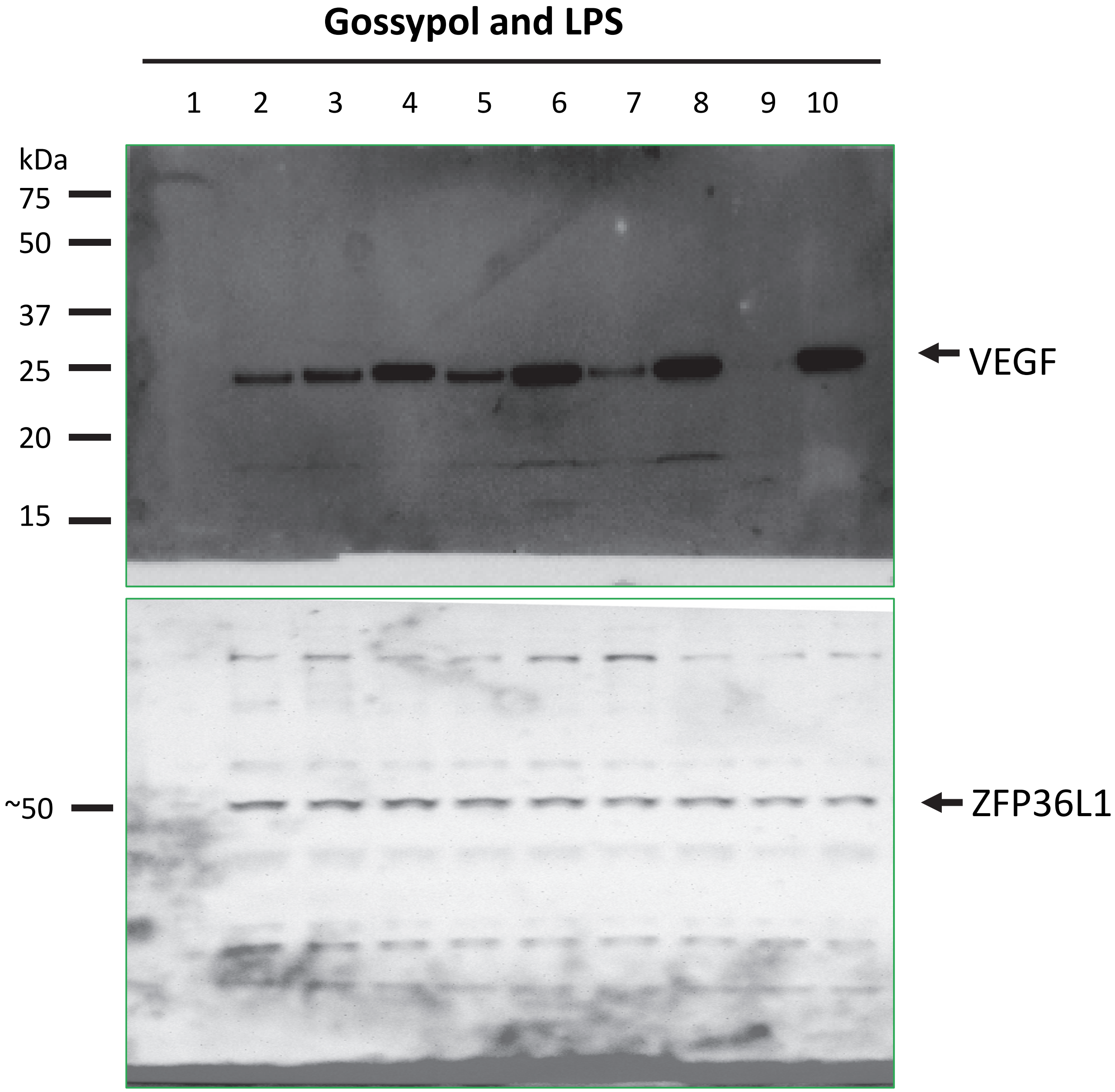

Fig. 6 Original Blots for Gossypol and LPS on VEGF and ZFP36L1 proteins
